# PROTAX-Sound: A probabilistic framework for automated animal sound identification

**DOI:** 10.1371/journal.pone.0184048

**Published:** 2017-09-01

**Authors:** Ulisses Moliterno de Camargo, Panu Somervuo, Otso Ovaskainen

**Affiliations:** 1 Department of Biosciences, University of Helsinki, Helsinki, Finland; 2 Department of Biology, Norwegian University of Science and Technology, Trondheim, Norway; Universidad de Salamanca, SPAIN

## Abstract

Autonomous audio recording is stimulating new field in bioacoustics, with a great promise for conducting cost-effective species surveys. One major current challenge is the lack of reliable classifiers capable of multi-species identification. We present PROTAX-Sound, a statistical framework to perform probabilistic classification of animal sounds. PROTAX-Sound is based on a multinomial regression model, and it can utilize as predictors any kind of sound features or classifications produced by other existing algorithms. PROTAX-Sound combines audio and image processing techniques to scan environmental audio files. It identifies regions of interest (a segment of the audio file that contains a vocalization to be classified), extracts acoustic features from them and compares with samples in a reference database. The output of PROTAX-Sound is the probabilistic classification of each vocalization, including the possibility that it represents species not present in the reference database. We demonstrate the performance of PROTAX-Sound by classifying audio from a species-rich case study of tropical birds. The best performing classifier achieved 68% classification accuracy for 200 bird species. PROTAX-Sound improves the classification power of current techniques by combining information from multiple classifiers in a manner that yields calibrated classification probabilities.

## Introduction

Non-invasive animal sampling techniques such as automated audio recording offer a powerful alternative to traditional capturing methods, with fewer adverse implications for wildlife welfare and reduced sampling biases [1]. Numerous types of field-optimized recorders (lightweight, waterproof, wireless and with large memory capacities) are commonly used in ecology, allowing to integrate large amounts of information across multiple spatial, temporal and biological scales. This poses key challenges for audio analysis, the most obvious being that with increasing amount of data, manual processing becomes unfeasible, and thus either drastic subsampling or automatic identification is necessary.

Virtually all vocal species have unique acoustic patterns that differ significantly among species, yielding a natural tag that allows for population monitoring [2]. Animal vocalization can be used to obtain both life-history information (*e*.*g*. sex or behavior) and ecological information (*e*.*g*. abundance, habitat use, survival, immigration and emigration). At least in theory, entire vocal species communities can be automatically identified from data provided by autonomous recorders. However, in practice it has been challenging to develop automated identification algorithms that would reach even close to the same level of species identification as obtained by manual identification by experts.

There is vast literature about automated species identification, including applications for insects, bats, whales, amphibians and birds [3]. A multitude of technical alternatives has been developed both for sound processing and pattern recognition tasks [4]. With the development of cutting-edge methods in machine learning [5] and the continuous growth of computational power, the set of available options is rapidly increasing. The bioacoustics community has recently started to systematically compare different techniques by applying them to the same datasets (e.g., the bird species identification challenge LifeCLEF [6]). Not surprisingly, as animal vocalizations vary broadly, some methods have been found to perform well for some groups but poorly for others. Additionally, existing classification methods give good results if the target classes are well represented in the reference database, but it has remained difficult to conduct reliable identifications if the reference database is sparse. With any method of species identification, a central question concerns the reliability of the classification. To move from sound similarity to a more objective measure of the reliability of species identification, the fullest solution would be to estimate the entire set of probabilities by which the query sound represents the possible candidate species. Among the existing classifiers, only few yield robust probabilistic output, and the reliability of such output has not been systematically evaluated [7].

In our previous work, we have developed a probabilistic method for taxonomic classification (PROTAX) of DNA sequence data [8–10]. PROTAX is a statistical model that estimates the probabilities by which the best matching reference sequences represent the species behind the query sequence. The probabilistic classifications by PROTAX take into account not only the species for which reference sequences are available, but also species that are known to exist but for which no reference sequences are available, as well as species–or higher taxonomic units–that are not known to science in the sense that they are missing from the taxonomy. PROTAX also accounts for the possibility that some of the reference sequences are mislabeled, a complication often present with DNA data. In this paper, we modify PROTAX to work with sounds rather than with DNA sequences, to present a method for probabilistic classification of animal sounds, called PROTAX-Sound. We use a species-rich case study of tropical birds to illustrate how the statistical framework is able to perform automated species classification in an accurate manner. In particular, we show how PROTAX-Sound can improve the classification accuracies of other classifiers and calibrate their estimates of classification uncertainty. We provide code and instructions to allow users to process their own audio with PROTAX-Sound.

## Materials and methods

### The PROTAX-Sound framework

**Fig 1:**
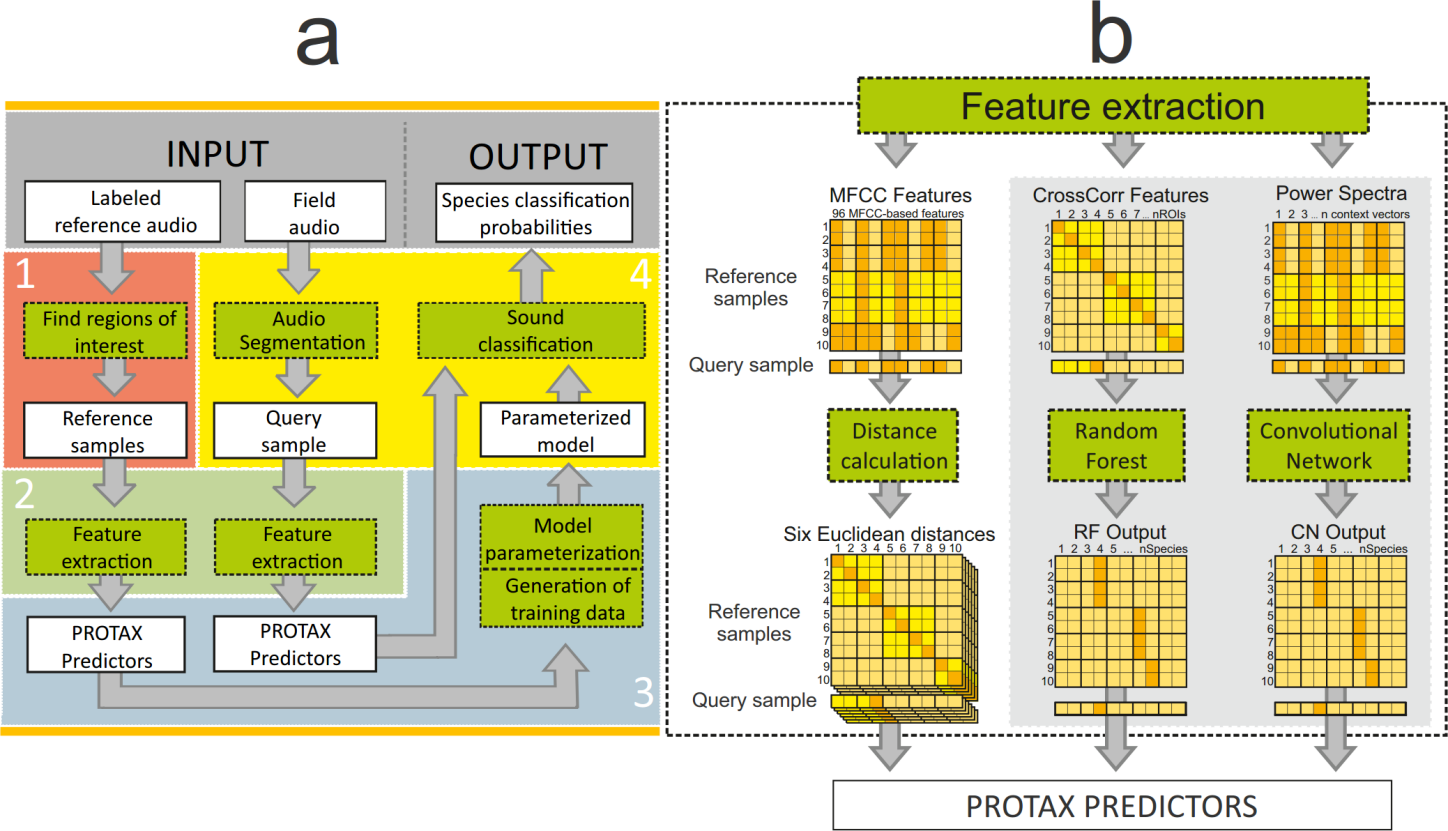
A schematic overview of PROTAX-Sound, a probabilistic classification system for animal sounds. Input files consist of labeled reference audio and field audio to be classified. The final outputs are the predicted classification probabilities for segments of field audio. Green boxes represent PROTAX-Sound functions; white boxes are inputs and outputs of these functions. The acoustic features and PROTAX-Sound predictors are calculated in the same way for both reference and query samples. The distances calculated from the MFCC features are used as PROTAX-Sound predictors. The cross-correlation features are used as input in the random forest model, the output of which is used to calculate PROTAX-Sound predictors. Mel-scaled log-power spectra of selected frames are used as input in the convolutional neural network, the output of which is used to calculate PROTAX-Sound predictors in the same way as for random forest. Panel a) shows the overall framework and panel b) the feature extraction pipeline (box 2 in panel a) in more detail, illustrated with MFCC features, cross-correlations features classified by Random Forest and power spectra features classified by convolutional neural network.

The overall workflow of PROTAX-Sound identification system has four phases: 1) the construction of a reference database consisting of manually identified vocalization samples; 2) the definition of acoustic features to be used to perform the classification; 3) the parametrization of the classifier; and 4) the extraction of query samples from field recordings and their classification (Fig 1). Steps 1–3 need to be done only once for a given set of reference species, while step 4 is repeated for each field audio to be scanned. The output from PROTAX-Sound is the predicted classification of each query sample, which in its fullest version is the vector of probabilities for all possible outcomes implied by the reference database. In the following sections, we describe the pipeline of Fig 1 in more detail.

#### 1) Construction of the reference database

Ideally the reference database includes several samples of all relevant types of vocalizations of all species present in the study area, recorded using the same equipment and under similar conditions as the field recordings to be identified. Besides the samples of the target species, the reference database may include as outgroups vocalizations of other species and background noise.

**Fig 2:**
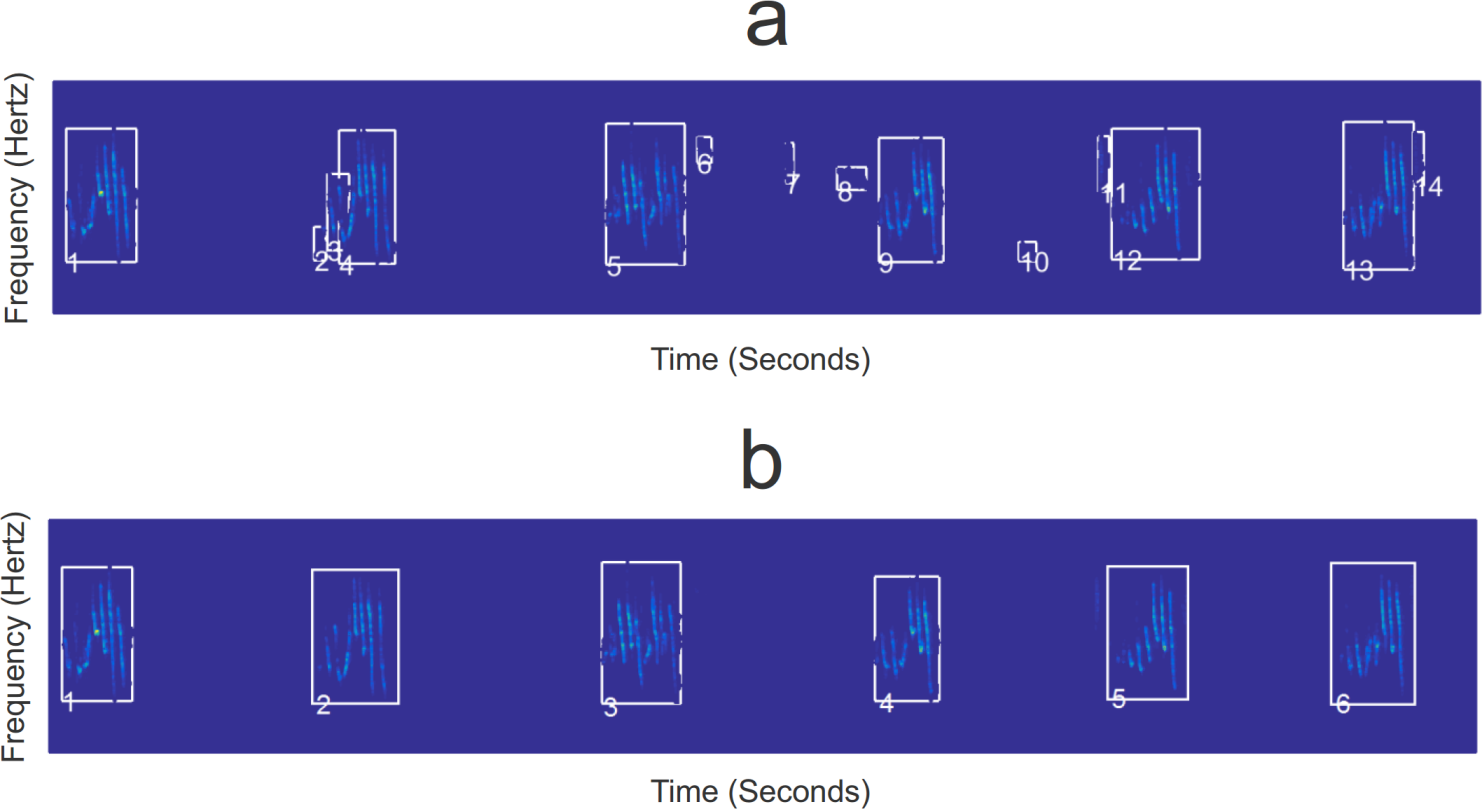
The regions of interest (ROI) extracted from the spectrogram image of a reference audio file. Panel a) shows ROIs suggested by an automated algorithm, including also ROIs that do not belong to the target species (*i*.*e*., background noise) or that should be merged. Panel b) shows the results after a manual validation phase.

To compile the reference database from audio files (*e*.*g*. WAV files from field recordings, online libraries or collection CDs), regions of interest (ROI) can first be located using unsupervised extraction of templates [11–14]. In brief, the template method (see supporting information S1 Text for details) consists of a sequence of image processing techniques performed over the spectrogram image of the sound. The automatically identified ROIs can optionally be validated manually, meaning that the user may visually check the quality of the segmentation, and redraw or delete unwanted ROIs when necessary (Fig 2). In the end, each sample in the reference database consists of the coordinates for the ROIs in the audio file, and the known species identity.

#### 2) Defining acoustic features

In the same way as PROTAX can classify DNA sequences based on any kind of sequence similarity measures or output of other classifiers [9], PROTAX-Sound can classify sounds based on similarities between any kind of numerical features extracted from audio samples. To illustrate PROTAX-Sound, we have selected to use as predictors (i) the Mel-frequency Cepstral Coefficients (MFCCs), (ii) the output of the Random Forest classifier based on the cross-correlation between the query sounds and the reference ROIs [14, 15], and (iii) the output of the Convolutional Neural Network classifier. MFCCs are the most widely used features in both speech recognition and animal sound identification, whereas cross-correlation based features have performed very well in bird sound classification challenges [14], and Convolutional Neural Network classifiers represent a newly emerging method. For a discussion about some alternative choices, we refer to [16] and [17].

We computed 16 MFCCs for each 0.02 second long frame that make up the ROIs of a given audio sample (see supporting information S1 Text for details). To obtain information about the temporal variation of the MFCC coefficients, we also computed the so called Delta and Delta-Delta features [18] for each MFCC coefficient, resulting into 48 parameters per frame. We then computed the mean and variance of each of these parameters over the frames of all ROIs that make up the audio sample, resulting into 96 parameters per sample (Fig 1B). We computed the dissimilarity between two samples as the Euclidean distance between the MFCC feature vectors, resulting into six dissimilarity values corresponding to the mean and variance for the three MFCC-based features.

We computed the normalized cross-correlation between the query sounds and the reference ROIs by sliding the spectrogram images of these two across each other and identifying the position that results in highest correlation of signal amplitude (see supporting information S1 Text for details; [19]). The values range from 0 to 1 for completely uncorrelated to completely correlated (or identical) images. We utilized the correlation values between a single query sample and all reference ROIs as predictors in a Random Forest (RF) classification algorithm (see supporting information S1 Text for details), and used the classification probabilities provided by RF as predictors for PROTAX-Sound (see below, Fig 1B).

As an alternative to the RF classifier, we used the Convolutional Neural Network classifier (CN), for which we computed predictors by processing the raw audio data into spectrograms by calculating Mel-scaled log-power spectra at every 10ms (see supporting information S1 Text for details). We used the classification probabilities provided by CN as predictors for PROTAX-Sound (see below, Fig 1B).

#### 3) The parameterization of the classifier

The PROTAX approach classifies samples against a hierarchical classification tree, which can be constructed independently of the reference database and can thus consist also of species that are not included in the reference set [9]. PROTAX predicts probabilities using a multinomial logistic regression model which decomposes the probability of one among all possible outcomes [9], which approach can be viewed as an application of the Platt scaling method [20]. In case of DNA-based molecular species identification, it is natural to use a taxonomical tree as the classification tree, in which case the classifier will conveniently yield probabilistic classification to the levels of species, genera, orders, and so on. However, as vocalizations reflect taxonomy only to a limited extent, we choose to use PROTAX-Sound without a hierarchical tree structure.

Like the DNA classifier PROTAX, also PROTAX-Sound can utilize any kind of numerical predictors. Here we illustrate its performance with the following eight predictors. We calculated six MFCC-based dissimilarities (see *Defining acoustic features*) between the query sample and all reference samples under the putative species. The predictors 1–6 are the minimum value of these dissimilarities over the reference samples available for the species. The predictor 7 is the logit-transformed class probability *p* assigned for the query sample by the RF classifier. The predictor 8 is the logit-transformed class probability *p* assigned for the query sample by the CN classifier. For predictors 7 and 8, in order to avoid singular cases related to *p* = 0 or *p* = 1, we modified the usual logit-transformation $logit(p)=log\left(\frac{p}{1-p}\right)$ to logit(*ε* + (1 − 2ε)*p*), where we set ε = 0.001. In addition to classifying the query samples as one of the species existent in the database, PROTAX-Sound also accounts for the possibility that the species behind the query sample may be outside the reference database, in which case the correct classification should be ‘unknown species’. We estimated the model parameters using the method of [9]. We thus generated training data by considering one reference sample as a query sample, and computing the predictors of PROTAX-Sound for this query sample. To mimic the case of a species included in the classification tree, we simply removed the query sample from the reference database to avoid circularity. To mimic the case of unknown species, we modified the classification tree by removing the leaf representing the species behind the query sample and all reference samples associated to it.

#### 4) Scanning and classification of field recordings

To classify sounds from continuous field recordings PROTAX-Sound first scans through the field audio files and locates candidate sounds to segment. The candidate sound regions form the query samples are filtered for background noise elimination (see supporting information S1 Text for details) and further processed for feature extraction and predictor construction (see *Defining acoustic features* and *The parameterization of the classifier*). In the end the query sample is classified by using the parameterized model to predict probability values for each possible outcome based on the reference set of species.

In the present study, however, we utilized the entire audio track as the query sample to be classified. This was done because the test audio data are not continuous field recordings with long periods of silence and noise-only regions, thus no further processing for locating candidate sounds was necessary.

### Increasing computational and statistical efficiency with feature pre-selection

It can be computationally and statistically inefficient to run PROTAX-Sound for all MFCC and cross-correlation features that can be computed for a large reference database. We thus aimed to filter out redundant, spurious and noisy reference ROIs, the inclusion of which may not only increase computational time but also decrease the accuracy of the classification. We selected the most informative ROIs based on the importance of the features as outputted by the Random Forest model fitted to the cross-correlation features (see supporting information S1 Text for details; [21]). We started with a Random Forest model trained for all cross-correlation features and then retrained it with increasingly smaller number of features, always using the most important ones. We chose the optimum amount to be selected based on the configuration that showed highest classification accuracy.

### Evaluating the performance of PROTAX-Sound: A case study on tropical bird vocalizations

For the evaluation of the PROTAX-Sound method, we used as a case study tropical bird vocalizations extracted from the Xeno-Canto collaborative sound library (http://www.xeno-canto.org). This is part of the same dataset used in the Bird task of LifeCLEF classification challenges, enabling us to compare to classification results by other methods. The dataset we used comprises the 200 tropical bird species most numerously represented in Xeno-Canto, gathered from field sites in Brazil, Colombia, Venezuela, Guyana, Suriname and French Guiana [6]. Audio files are stereo and recorded at sampling rate of 44,100Hz, with generally good quality but with variation in the level of noise due to *e*.*g*. weather conditions and the amount of background species, as common when building reference databases from heterogeneous sound sources.

Based on the metadata provided by Xeno-Canto, we selected species represented by at least five audio files, and constructed the database only with maximum quality files (class 1) which had no background species. We used a five-fold cross-validation strategy by dividing audio files in five groups to be used as training and test data, i.e. we used four folds to training and the remaining fold for classification, and iterated until all audio tracks had been classified. This strategy guaranteed that we always used independent data for training and for testing our approach. The species were divided between folds in the most balanced way and all folds had at least one audio file for all species. Multiple recordings for the same species were considered to possibly represent the same individual if recorded by the same author and on the same day [22], and such cases were never included in both the training and classification groups to ensure the independence of the classification data.

We assessed the classification performance of four different PROTAX-Sound versions: PROTAX-Sound (MFCC+RF+CN), which utilized all sets of predictors listed above, as well as PROTAX-Sound (MFCC), PROTAX-Sound (RF) and PROTAX-Sound (CN) that included only one of the sets of predictors. The motivation for this was to examine how much classification resolution each set of predictors provides, as well as to examine if they provide complementary information (*i*.*e*., if the joint model performs better than any of the three models alone). In addition, we assessed the classification performance of the Random Forest and the Convolutional Neural Network classifiers directly instead of using them as predictors for PROTAX-Sound. The motivation for this was to examine if the classification probabilities of Random Forest and Convolutional Neural Network are not calibrated, and if yes, whether the PROTAX-Sound approach can calibrate them.

Ideally, a classifier does not give as output just the most likely class, but the probability by which the query sequence belongs to any candidate class. To evaluate the classification accuracy and to examine whether the classification probabilities are calibrated, we mimicked a user who would pick up the class that received the highest probability. To assess the accuracy of these classifications, we examined which fraction of them belonged to the correct species. Before describing how we assessed if the classification probabilities are calibrated, let us first note how they should behave if they are calibrated. As an example, assume that there are 100 query samples for which the highest classification probability is 0.8 (and consequently, the remaining classification probabilities sum to 0.2). In this case, the species corresponding to the highest classification probability should be the correct species in 80% of the cases, whereas it should not be the correct species in 20% of the cases (hence, in these cases the correct species is one of the candidate identifications with a lower classification probability). If this is not the case, then the classification probabilities are not calibrated, being either under- or overconfident. While under-confident classification probabilities may be preferred over overconfident ones, clearly it is most preferable to have calibrated classification probabilities and thus a reliable assessment of identification uncertainty. To examine for the presence of a bias, we used reliability diagrams [23]: We ordered the highest identification probabilities from the lowest to the highest, and paired with each the information of whether the classification was correct (1) or not (0). We then plotted against each other the cumulative sums of the identification probabilities and the numbers of correct classifications. If the highest classification probabilities are calibrated (and separately so for small and large probabilities), such a plot will follow the identity line, whereas a deviation from the identity line will indicate uncalibrated values.

Even if the identification probabilities would be on average calibrated, they could still be under- or overconfident for some individual species. To examine for such a possibility, we repeated the above described procedure separately for each focal species. Instead of plotting the results as we did for the overall summary, we generated for each species an empirical p-value that describes the level of bias in the identification probabilities. To do so, we simulated the null distribution for the number of correct outcomes for each species by summing the results of *n* Bernoulli trials related to the highest probabilities predicted by PROTAX-Sound for each of the *n* samples belonging to the species. This process was repeated 10,000 times to generate the null distribution. We then calculated a p-value based on a two-tailed test to check whether the observed number of correct classifications deviates from the simulated numbers. A small p-value implies that the probabilities estimated by PROTAX-Sound would have a very low chance of producing the observed outcome for the species, indicating that these probabilities are not calibrated.

## Results

**Fig 3:**
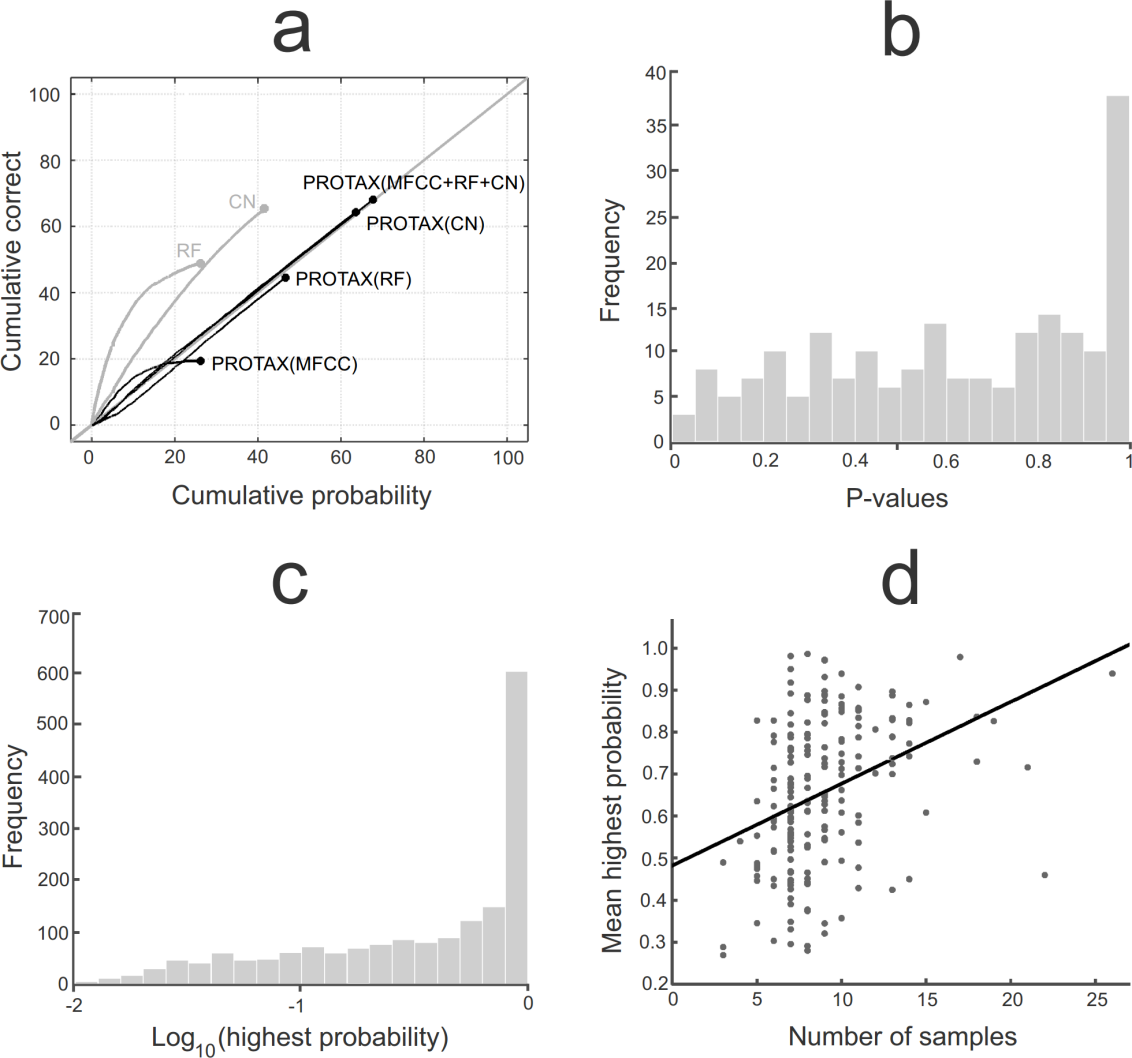
Accuracy and bias of 1766 test samples identified by different versions of PROTAX-Sound, Random Forest and Convolutional Neural Network classifiers. Panel a) shows reliability diagrams for the best outcome species (x-axis) and the cumulative correctness of the prediction (y-axis). The six lines correspond to the raw output of Random Forest (RF), the raw output of Convolutional Neural Network (CN) and the PROTAX-Sound models that use MFCC, RF, CN or their combination as predictors. The model-predicted probabilities are calibrated if the lines follow the identity line (the grey diagonal line), and they are the more accurate the higher the lines reach. Panel b) shows the distribution of p-values for the 200 species classified by PROTAX-Sound (MFCC+RF+CN), asking if the classifications are not calibrated for some particular species. Panel c) shows the distribution of the highest PROTAX-Sound (MFCC+RF+CN) probabilities predicted for each of the test samples. Panel d) shows the highest PROTAX-Sound (MFCC+RF+CN) probability against the number of reference samples. In this panel, each dot corresponds to each of the 200 species, and the probabilities are averaged over all test samples that belong to the species.

The performance of PROTAX-Sound against the 1766 test samples is shown in Fig 3. The best model version was PROTAX-Sound (MFCC+RF+CN) that combines outputs from the MFCCs, Random Forest and Convolutional Neural Network, for which the classification with highest probability was the correct species in 1203 cases (68% accuracy). The predicted classification probabilities were generally calibrated, as the line depicting the relationship between predicted and true identities falls very close to the identity line in Fig 3A. The second best performance was achieved by the Convolutional Neural Network alone which reached 65% accuracy, but with substantial bias on its predicted probabilities: the probabilities outputted by the Convolutional Neural Network suggested a 41% accuracy. The Random Forest algorithm alone reached 48% accuracy, but also with substantial bias in its predicted probabilities: the probabilities outputted by the Random Forest algorithm suggested only 26% accuracy. For both cases the CN and RF classifiers, PROTAX-Sound was able to convert the biased classification probabilities into calibrated ones, thus reducing their bias (Fig 3A). PROTAX-Sound parameterized with the MFCC predictors achieved only 19% accuracy, showing that the MFCC features have much poorer classification power than the cross-correlations utilized by the RF and CN algorithms.

The species-specific assessment of PROTAX-Sound (MFCC+RF+CN) showed that also the species-specific probabilities are well calibrated. If they were fully calibrated, the histogram of species-specific p-values generated by the null-model approach should be uniform in the range [0,1]. As shown in Fig 3B, this is close to be the case, except the peak near p = 1, which is due to the discreteness of the distribution for small sample sizes and can thus be ignored. Thus, there is no evidence for uncalibrated probabilities even in the species-specific identification probabilities.

**Fig 4:**
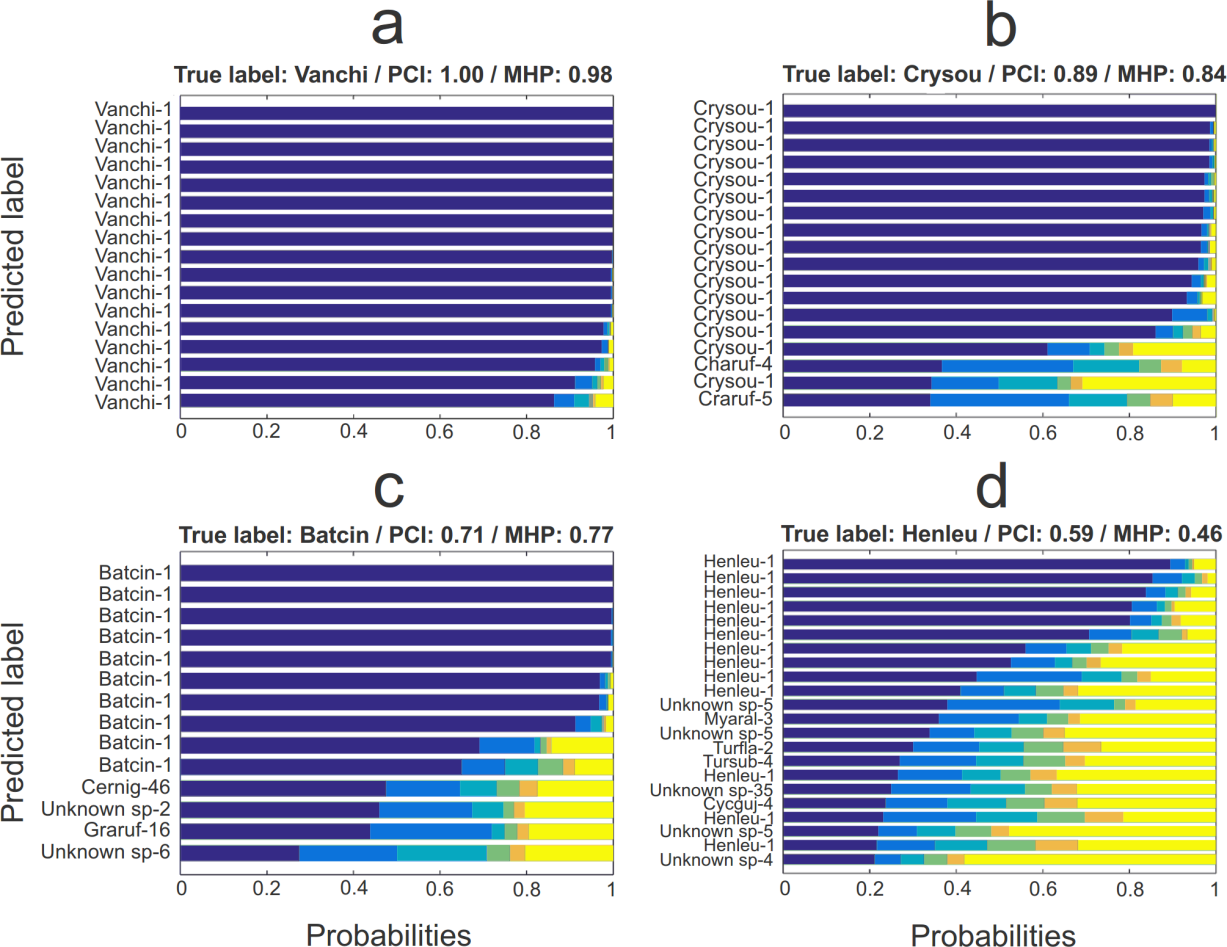
Illustration of the performance of PROTAX-Sound (MFCC+RF+CN) for selected example species. Each panel corresponds to one focal species, and each bar corresponds to a single query sample originating from that focal species. The colors summarize the predicted probability distribution of species identity over all species in the reference database, presented in descending order of probabilities. The five highest probabilities are shown in distinct colors, whereas the remaining probabilities are summed together and shown by the yellow bar. The predicted label is the species that PROTAX-Sound assigned the highest probability (the dark blue part of the bar), and the number after labels is the rank of the identification that corresponds to the true species. For each focal species is also shown the proportion of correct identifications (PCI; fraction of cases where the identity with highest probability corresponds to the true species) and the mean highest probability assigned by PROTAX-Sound (MHP; average over the highest probabilities, whether they represent the true species or not). The species have been selected to show contrasting cases of identification uncertainty: a) *Vanellus chilensis*; b) *Crypturellus soui*; c) *Batara cinerea*; d) *Henicorhina leucophrys*. For full names of all species and their abbreviations, see supporting information S2 Table.

Fig 4 illustrates the nature of the probabilistic identifications for four focal species, with variation in the certainty by which the vocalizations can be identified. More generally, the distribution of the highest identification probabilities (Fig 3C) involves much variation and reflects the range of difficulties in acoustic species identification that is faced also by an ornithologist conducting similar identifications manually: some vocalizations are very easy to identify while others are hard to distinguish from other similar vocalizations. Amongst the identifications with much uncertainty there were 139 samples for which PROTAX-Sound assigned the highest probability to ‘unknown species’, indicating that the similarity between the query sample and the best matching reference sample was not better than the matches between reference samples belonging to different species.

The variation among the species in their classification probabilities can be attributed to two sources. First, it is evident that species with distinct vocalization patterns lead to more confident identifications than is the case for species whose vocalizations are similar to some other species. To illustrate, let us consider the parrot *Ara macao*, with vocalizations very similar to other species within the Psittacidae family. For the test samples which represent the species, PROTAX-Sound assigns on average only 9% of identification certainty to the correct species, but it is 84% sure that the samples represent one of the Psittacidae species (sum of the identification probabilities for the species within the Psittacidae family, see supporting information S1 Table). This example also illustrates that PROTAX-Sound can be used to estimate identification probabilities not only for individual species but also for groups of species. Second, the number of reference samples correlates positively with classification accuracy (Fig 3D), most likely due to the fact that the likelihood of the inclusion of a high-quality reference sample which matches well with the query sample increases with the number of reference samples.

**Fig 5:**
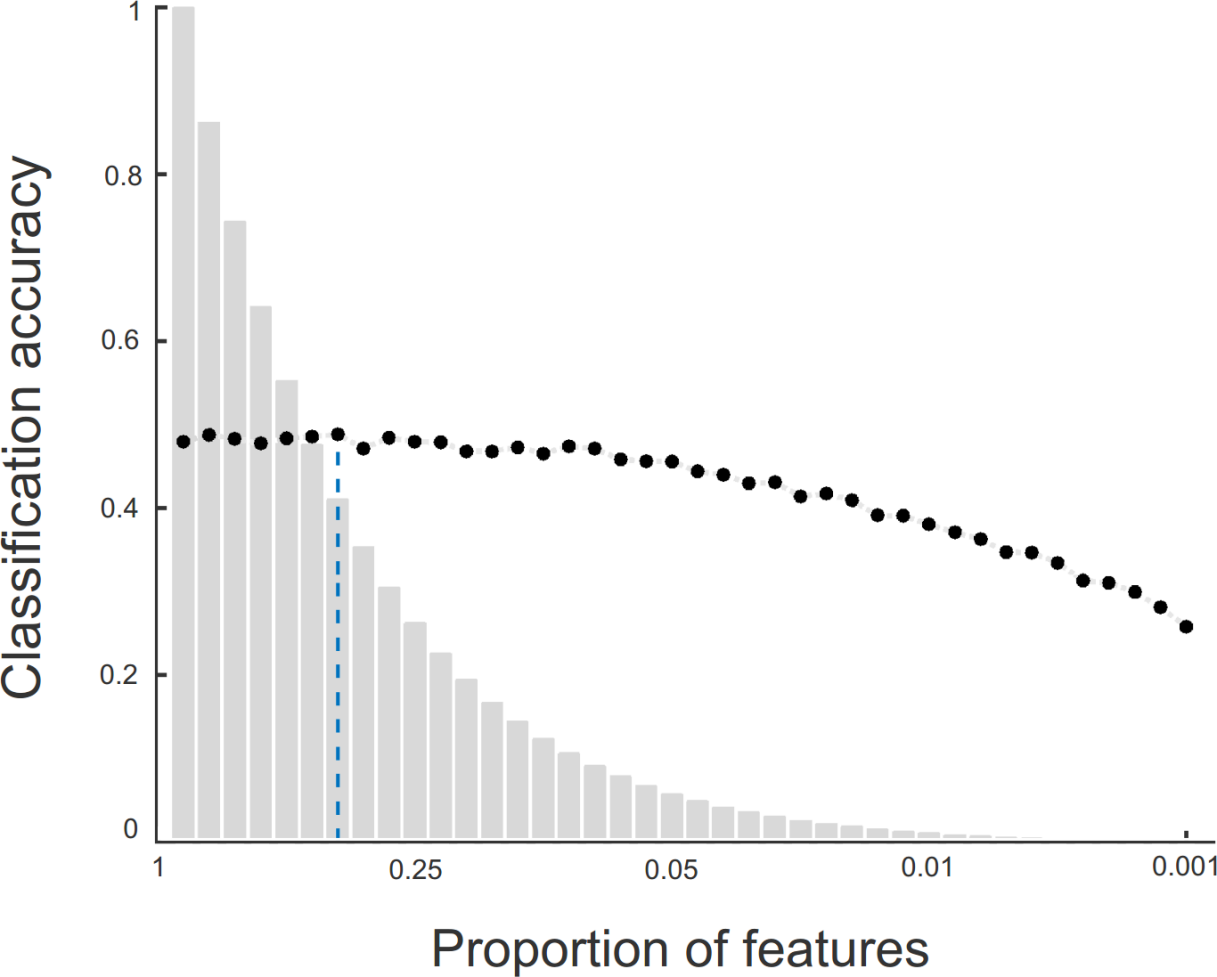
Gain in computational and statistical efficiency due to feature pre-selection. The classification accuracy for the Random Forest algorithm (y-axis; black dots) was calculated over the training data and for different amounts of cross-correlation features (x-axis; grey bars). The choice of the quantity of features to be used as PROTAX-Sound predictors was based on the configuration which showed the highest classification accuracy based on as few features as possible (dashed blue line).

The feature selection experiment showed that including only approximately 40% of the most important features improves the accuracy of the Random Forest algorithm from the 46% baseline (model with all features) to 49% (Fig 5). Besides the slight gain in accuracy, reducing the number of features significantly decreases the total computational time when classifying field audio data.

## Discussion

In this work, we have utilized recent developments in probabilistic taxonomic classification methods for DNA sequences to develop a PROTAX-Sound, a statistical framework for probabilistic species identification for audio samples. We have demonstrated that PROTAX-Sound is able to convert similarities of audio features into calibrated species membership probabilities. The estimated probabilities reflect the same intuitive evaluation of uncertainty used by human experts [24], with PROTAX-Sound leaving much uncertainty for species with similar vocalizations, or species that are poorly represented in the reference database. Like a human expert encountering a new vocalization, PROTAX-Sound assigns a high probability for the *unknown species* class when encountering a query sample which does not share similarity with any of the species in the reference database. As illustrated by our example of the parrots within the Psittacidae family, sometimes PROTAX-Sound, or equally a human expert, can make a confident identification only at the level of a group of species, not at the level of an individual species.

Random Forest is increasingly used in automated sound classification due to its good accuracy and various implementation advantages: it is fast, scalable and robust to noisy and correlated data [22, 25–27]. However, as we illustrated here (Fig 3), the identification probabilities predicted by RF are not necessarily calibrated and can thus substantially compromise the robustness of biological inference derived from species classifications. In our study the CN approach was the best performing algorithm, but also it provided non-calibrated identification probabilities. In contrast, the identification probabilities predicted by PROTAX-Sound (RF) or PROTAX-Sound (CN) were calibrated, making the assessment of species identification uncertainty reliable and ready to be propagated to downstream analyses. Thus, when using the output of a single classifier as a predictor, as we done here with RF and CN, PROTAX-Sound can be viewed as a statistical wrapper to calibrate the probabilities outputted by the classifier (but see [28] for alternatives).

Our results show that for some species there remained much uncertainty (Fig 4), and thus additional predictors are needed to improve accuracy. Importantly, PROTAX-Sound allows one to use as predictors any combination of audio similarity measures and classifiers. Ideal predictors have good classification resolution (*i*.*e*., have much variation among species but only little within a species) and provide complementary information. In order to further develop potentially complementary predictors, we note that the temporal component of bird sound provides important information for discrimination, *e*.*g*. calls, chirps and warbles having different temporal patterns [29]. We attempted to incorporate temporal variation by using the delta coefficients extracted from MFCC [30], but the poor results from PROTAX-Sound (MFCC) suggest that these features failed to capture the relevant parameters. One possibility is to explicitly model the temporal variation of the acoustic signal by considering *e*.*g*. the order in which the ROIs appear in a track, their lengths, and the duration of gaps between them. Such data can be used as input information for Hidden Markov Models [31, 32], the output of which could be used as a predictor for PROTAX-Sound, potentially providing complementary information from other predictors such as RF or CN used here. PROTAX-Sound (MFCC+RF+CN) being the best accuracy model emphasizes the power of complementary predictors to achieve best classification results.

PROTAX-Sound classifies query samples not only for the most likely species, but for the full set of species present in the reference database, and can generate detection matrices of query samples times the probability of placement for all target species. When building *e*.*g*. Bayesian models of bird community dynamics [33], or joint-species distribution models [34], the collection of such detection matrices can be considered as a prior for the true occurrence matrix. Then one can sample the posterior distribution of the true occurrence matrix, thus enabling to propagate species identification uncertainty through the community modeling analyses.

Statistical methods with great potential for automated identification are continuously appearing in the scientific literature [35], and as discussed above, PROTAX-Sound provides a statistically rigorous method to combine the strengths of the different techniques. In parallel with the development of statistical methods, it is important to make the methods applicable to real data acquired from field conditions [36]. One practical challenge with automated processing of audio samples is generated by variation in the amount and type of background noise [37–39]. Among the multiple methods developed for filtering out background noise [40–42], we adopted the simple but effective median filtering technique to remove background noise (see supporting information S1 Text for details), successfully applied in recent automated identification studies [22, 43]. The technique performs very well for audio with relatively constant background noise (e.g., white noise caused by moderate wind), which was the case for the majority of the data we classified. This might not be the case for more difficult recording conditions (e.g., equipment near to streams or in windy areas) and then extra filtering may be needed.

While we have illustrated the use of PROTAX-Sound specifically for identifying bird sounds, it provides a general framework to classify the sounds of any vocal animals, such as bats or frogs. While there are still undoubtedly further challenges associated with improving the classification accuracy of the method, and in applying it to noisy and heterogeneous field recordings, we hope that the framework developed here provides a robust starting point for probabilistic identification of animal sounds, making it possible to propagate the unavoidable uncertainty in species identifications to biological inference derived from audio data.

## Supporting information

S1 Text Methods appendix. Detailed description of audio processing and classification algorithms.
(DOCX)

S1 Table Results appendix. Table containing the 1766 sample-specific full output of PROTAX-Sound classification results.
(ZIP)

S2 Table Species names and IDs. Latin names and 6-letter short names for 200 study species.
(TXT)

S3 Table 5-fold data tags. Logical table containing the 5-fold division of the 1766 audio files used for training/test of PROTAX-Sound.
(TXT)

S4 Table Xeno-Canto ID per sample. 1766 unique identifiers used to locate each of the used audio files in Xeno-Canto database.
(TXT)

S1 Code PROTAX-Sound functions. Zip file containing all code necessary to run PROTAX-Sound.
(ZIP)
