# Supplementary material for: PROTAX-Sound: A probabilistic framework for automated animal sound identification
Source: PLoS One. 2017 Sep 1;12(9):e0184048. doi: 10.1371/journal.pone.0184048 (PMC5581177; doi:10.1371/journal.pone.0184048)
Supplement: S1 Text — Detailed description of audio processing and classification algorithms. (DOCX) [file pone.0184048.s001.docx]

**Supplementary information**

**2) Unsupervised extraction of Regions of Interest (ROI detection)**

The unsupervised method we applied to extract ROIs to be used as templates is fully described in the work by [1] and it has been successfully applied in many previous work, as reviewed by [2]. In short, a given 44.100Hz audio track is first down sampled to 22.050Hz and Fast Fourier transform (FFT) is applied by using a Hanning window with a size of 512 samples and 75% overlap, followed by the normalization between zero and one of the resulting amplitude values. The spectrogram of each audio file is then treated as an image with frequency in the y-axis and time in the x-axis, and it is further processed for background noise reduction and ROI segmentation.

The background noise is greatly reduced by first applying a Gaussian blur to the sonogram image, followed by median clipping and its subsequent processing by image processing techniques (closing, dilation, and median filter;[3]). The median clipping consists of generating a binary image where each pixel value is set to 1 if its amplitude is above *n* times the median of its corresponding frequency axis and *n* times the median of its corresponding time axis, otherwise it is set to 0 (for good quality reference audio *n* = 3 showed to be a good threshold). Lastly, all connected pixels with value 1 that exceeds a certain minimum size (500 pixels in this study) are labeled as a ROI and a numbered rectangle is used to define its size and position (Fig. 2). The entire process is computationally fast and successfully finds ROI corresponding to vocalizations for most of the species. At the end of the ROI construction task there is a manual validation step, where users may visually check the quality of segmentation and redraw or delete unwanted ROI when necessary (see *PROTAX-Sound Code and results reproducibility*).

**3) Feature Extraction: Mel-Frequency Cepstral Coefficients (MFCC)**

MFCC were first described by Davis and Mermelstein in 1980 [4]. The implementation we used in our Matlab code was created by Ellis in 2005 [5] and the detailed settings are specified within *PROTAX-Sound_functions.zip*. MFCC are derived from a type of audio representation that approximates the way sounds are perceived by the human auditory system (*i.e.*, the Mel-scale). In short, the original audio is first framed to 0.02 second long window, then transformed to the frequency domain (by using discrete Fourier transform) and mapped into the Mel-scale. We then take the logarithm of the power spectral values and use the discrete cosine transform (DCT). The application of DCT results in the set of MFCC from which we utilize the first 16 coefficients. The first MFCC represents the sum of all the log-energies and is therefore an overall measure of sound loudness. Note the MFCC feature vector describes only the power spectral envelope of a single frame, but animal sound also carry temporal information *i.e.* the trajectories of the MFCC over time may also bring useful information for species classification. To capture that information we have calculated the Delta and Delta-Delta features. The delta coefficients are calculated as the difference between the MFCC from the current and the previous frame. Delta-Delta coefficients are calculated in the same way, but using the Delta coefficients values instead of MFCC values. To produce the final feature vector of a given query sample we just take the mean and variance of MFCC, Delta and Delta-Delta features over the frames.

**4) Feature Extraction: Cross-correlation based features**

The normalized cross-correlation is a widely used metric to compare templates for their similarity [6]. Fodor [7] was the first to apply cross-correlation in automated bird sound identification systems, and we also refer to [8] and [1] as key readings. The method is simple but can be time consuming, especially when the reference database contains a huge quantity of ROIs to be correlated against the query samples (but see “*Increasing computational and statistical efficiency with feature pre-selection”*). We computed the normalized cross-correlation between the query samples and all the reference ROIs by sliding the spectrogram images of these two across each other and identifying the position that results in highest correlation of signal amplitude. In order to have fewer cross-correlation calculations, instead of covering the entire spectrogram image of each query sample, the sliding of the templates is limited to the frequency range of the target ROI plus ±3 extra pixels as a buffer to account for small variations in the vocalizations. The vector containing the highest cross-correlation values for all ROIs against a given query sample is then used as input for the Random Forest. During the training phase this procedure is done by using the reference samples as the query samples, to generate training data for the Random Forest algorithm. During the classification phase the same procedure is applied, but using data from the test files as query samples.

**5) Random Forest classifier and feature selection experiments**

For a complete description of the Random Forest (RF) algorithm we refer to [9]. In short, RF fits many classification trees to a data set, and then combines the predictions from all the trees to get an improved classification. The algorithm starts with the selection of *e.g.* 400 bootstrap samples taken from the training data, each of it having approximately 63% of the original data. The remaining observations which were not selected are called out-of-bag observations and will be used later for validation and feature selection (see below). A classification tree is then fit to each bootstrap sample, and at each tree node, only a randomly selected fraction of the variables (*e.g.*, *n* =10) are available for the splitting. The trees are grown (*i.e.*, trained) and used to classify new test data, with class decision made by majority voting across the classifications of the entire forest.

As described in “*Increasing computational and statistical efficiency with feature pre-selection”*, we did feature selection experiments to guide how to filter out redundant, spurious and noisy ROIs from the reference database based on indexes of feature importance calculated with RF algorithm. RF has a simple way to calculate the misclassification rate of each tree in the forest, based on the classification of out-of-bag observations. To assess the importance of a specific predictor variable (*i.e.*, a specific feature value), the values of the variable are randomly permuted for the out-of-bag observations, and then the modified out-of-bag data are passed down the tree for classification. The difference between the misclassification rate for the modified and original out-of-bag data, divided by the standard error, is a measure of the importance of that variable for classification. We calculated these indexes using our training dataset, sorted the cross-correlation features from the more to the least important ones, and used it to select increasingly smaller number of features to be used in different RF experiments. The final configuration used to classify the test data was chosen based on the experiment which showed highest accuracy and used fewer features (Fig. 5).

**6) Convolutional Neural Network classifier**

In addition to random forest classifier, we computed another set of predictors using multilayer perceptron (MLP). We used Tensor-Flow [10] software with its Python API and adapted an exemplary code originally designed for MNIST handwritten digit recognition. This model consists of two convolutional layers with 5*5 input patches each followed by max pooling layer with 2*2 patches. The first layer has 32 and the second layer has 64 filters. Before the final output layer which has one node for each bird species class there is a fully connected layer with 512 units. Hidden layer units have ReLU activation functions and the output layer has Softmax activation function.

Raw audio was processed into spectrograms by calculating Mel-scaled log-power spectra at every 10ms. Original audio was resampled into 16 kHz, 256-point FFT was applied to overlapping 16ms Hanning windows and 32 Mel-bins were calculated from each frame covering frequencies between 500 Hz and 8 kHz. Record-specific mean was subtracted from logarithmic Mel-spectra and negative values were replaced by zeros. Adjacent time frames were averaged and concatenated within one second time interval to create a 32*32 dimensional context vector. Averaging was done so that the temporal resolution was most accurate in the middle of the window and most blurred at both ends. Context vectors whose central frame energy exceeded three times the median background energy were used as inputs to CN. In case none of the candidates exceeded the threshold, five context vectors with largest energy were selected per recording.

CN outputs from all context windows within a recording were summed and then scaled by the total sum of all nodes to give a probabilistic classification score.

**7) PROTAX-Sound Code and results reproducibility**

In this work, we have illustrated PROTAX-Sound by using data with known species labels collected from Xeno-Canto online library (www.xeno-canto.org). We provide unique identifiers to locate each of the used audio files in Xeno-Canto database (*xenocanto_idpersample.txt*) so readers can download the relevant audiofiles. To assure the reproducibility of results, all code we provide is described with the same settings as used to obtain our results. The functions and command pipelines are contained in the file *PROTAX-Sound_functions.zip*.

**7.1) Code dependencies and the Matlab functions**

There are two Matlab toolboxes which are needed for the PROTAX-Sound pipeline to run: The Parallel Computing Toolbox, which is applied to speed up computations, and the Statistics and Machine Learning Toolbox, which has the Random Forest algorithms. In addition, PROTAX-Sound utilizes m-files created by different authors: 1) The MFCC implementation made by Den Ellis, which is used for calculating the MFCC features (downloaded from http://www.ee.columbia.edu/~dpwe/resources/matlab/rastamat/rastamat.tgz); and 2) The Worker Object Wrapper made by MathWorks Parallel Computing Toolbox Team, which is used to simplify the managing of large data within parallel loops (downloaded from Matlab File Exchange).

**7.2) Instructions for using Matlab functions**

The analysis is divided in two parts: it starts with the Matlab file *predictorpipeline.m* which prepares the predictors for PROTAX-Sound; *enginepipeline.txt* involves Perl and R code (from [11]) and it contains the commands to model training and classification parts. The full results table is generated at the end of this pipeline. We now describe the Matlab functions in more detail:

**predictorpipeline.m** is the main script from which the other functions are called. It is divided in numbered steps that should run one at time until the end of the pipeline. Each step produces the input needed for the next step.

**roimap.m** is a function that finds ROIs in the reference audio and constructs the reference database. It is fully automated after inputs are provided. The output is called ‘roidata’, and contains all the information which will be used to extract audio features from ROIs.

**roival.m** is a function to validate or redraw ROIs in the reference database. Based on the visual inspection of figures saved by *roimap*, users can assess the quality of ROI detection and segmentation and decide which audio files need to be redrawn. *Roival* is not fully automated and further instructions are needed both before and during its use:

Users need to first create the ‘valdata.txt’ input file. This file lists information only for the audio files with ROIs that need to be corrected. The identifier for each audio file can be found as a number in the title of the figure generated by *roimap*. Those identifiers should be written in the valdata.txt as follows: each row but the last represents one audio file containing ROIs which should be deleted. The first number in a row is the identifier from the figure, followed by the number of the ROIs that will be deleted. The last row of the txt file brings the identifiers for audio files which ROIs need to be completely redrawn. All numbers are separated by commas and no delimiter is needed at the end of a row (Fig. S1).

When running *roival* users will be presented with a screen to draw ROIs manually, for each audio files listed in the last row of *valdata.txt*. During all the process users can resize the screen for a better visualization. Its use is simple: First draw a box around the target sound (note the box can be moved and resized). Double click the white box to allow mouse for drawing the second box (repeat this process to draw the following boxes until the last one). When finished, double click the last white box, then click the validate button and then double click the blue area of the screen two times. The next audio file in queue will load for redrawn. Repeat the process until all files are done. The process for files with ROIs to be excluded will be done automatically and new figures will be saved if sfig is set to 1.

When validating thousands of audio files, users may want to take a break, or computational problems may occur (*e.g.*, computer can crash). There are two input arguments used to avoid users from starting the drawing process from the beginning: *time2save* and *lastval*. Time2save will set the time to automatically save results after a number of audio tracks have been validated (when validating more than a thousand audio files, 50 showed to be a good number). Too small number will make the process slow (because of too frequent file savings) and too big number can be risky (if computer crashes user will need to start from the last saving point). Lastval is used to guide *roival* from where to start validation again after some error/break had occurred. If a break did occurred and one needs to restart, lastval value will be saved within roidatav.mat in the working directory; otherwise its value should be set to 1.

**featmfcc.m:** It calculates MFCC features from the reference or the query samples. Given the inputs, the function works fully automated and generates the MFCC output file.

**featccor.m:** It calculates Cross-Correlation features from the reference or the query samples. Given the inputs, the function works fully automated and saves cross-correlation output txt files in the working directory.


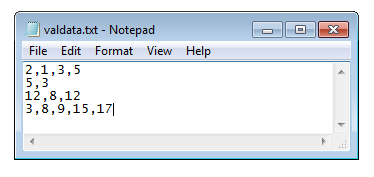


**Fig. S1.** Example of valdata.txt file. In this illustration, audio track 2 will have ROIs number 1, 3 and 5 deleted. Audio track 5 will have ROI number 3 deleted, and so on. The last row lists audio tracks that need to be redraw, *i.e.* tracks number 3, 8, 9, 15, and 17.

**References**

[1] Lasseck, M. 2015 Improved Automatic Bird Identification through Decision Tree based Feature Selection and Bagging. In The Cross Language Image Retrieval Track (CLEF) Conference (eds. L. Cappellato, N. Ferro, G. Jones & E.S. Juan). Toulouse, France, CEUR.

[2] Potamitis, I. 2015 Unsupervised dictionary extraction of bird vocalisations and new tools on assessing and visualising bird activity. Ecological Informatics **26**, Part 3, 6-17. doi:http://dx.doi.org/10.1016/j.ecoinf.2015.01.002.

[3] Shih, F.Y. 2009 Image processing and mathematical morphology: fundamentals and applications, CRC press.

[4] Davis, S. & Mermelstein, P. 1980 Comparison of parametric representations for monosyllabic word recognition in continuously spoken sentences. IEEE Transactions on Acoustics, Speech, and Signal Processing **28**, 357-366. doi:10.1109/TASSP.1980.1163420.

[5] Ellis, D.P.W. 2005 PLP and RASTA (and MFCC, and inversion) in Matlab. availeable at: http://www.ee.columbia.edu/ln/rosa/matlab/rastamat

[6] Lewis, J. 1995 Fast normalized cross-correlation. In Vision interface, pp. 120-123.

[7] Fodor, G. 2013 The Ninth Annual MLSP Competition: First place. In 2013 IEEE International Workshop on Machine Learning for Signal Processing (MLSP), pp. 1-2.

[8] Potamitis, I. 2014 Automatic classification of a taxon-rich community recorded in the wild. PLoS One **9**, e96936. doi:10.1371/journal.pone.0096936.

[9] Breiman, L. 2001 Random forests. Machine Learning **45**, 5-32. doi: 10.1023/A:1010933404324.

[10] Abadi M., Agarwal A., Barham P., Brevdo E., Chen Z., Citro C., et al. (2015) TensorFlow: Large-scale machine learning on heterogeneous systems. Software available from tensorflow.org. arXiv:1603.04467

[11] Somervuo, P., Koskela, S., Pennanen, J., Henrik Nilsson, R. & Ovaskainen, O. 2016 Unbiased probabilistic taxonomic classification for DNA barcoding. Bioinformatics **32**, 2920-2927. doi:10.1093/bioinformatics/btw346.
